# Dried blood spot is the feasible matrix for detection of some but not all hepatitis B virus markers of infection

**DOI:** 10.1186/s13104-022-06178-x

**Published:** 2022-09-05

**Authors:** Minami Kikuchi, Patrick Lindstrom, Alexandra Tejada-Strop, Tonya Mixson-Hayden, Saleem Kamili, Motoji Sawabe

**Affiliations:** 1 Division of Viral Hepatitis, Centers for Disease Control and Prevention, 1600 Clifton Road, Atlanta, GA 30329 USA; 2 Department of Molecular Pathology, Graduate School of Medical and Dental Sciences, Tokyo Medical and Dental University, 1-5-45 Yushima, Bunkyo, Tokyo 113-8519 Japan

**Keywords:** Dried blood spot testing, Hepatitis B surface antigens, Total hepatitis B core antibody, Hepatitis B e antigen, Hepatitis B virus DNA

## Abstract

**Objective:**

Use of dried blood spots (DBS) for detection of hepatitis B virus (HBV) markers of infection has the potential to facilitate diagnosis of HBV infection especially in resource-limited countries. The aim of this study was to evaluate the feasibility of DBS for detection of various markers of HBV infections.

**Results:**

Fifty-four DBS samples were engineered from well-characterized plasma samples. All DBS samples were tested for HBsAg, total anti-HBc and HBV DNA, 20 of 54 samples were also tested for HBeAg using commercially available assays. HBsAg was detected in 24 of 25 (96%), HBV DNA in 22 of 25 (88%), total anti-HBc in all 9 (100%), and HBeAg in all 7 (100%) DBS samples. The average difference in HBV DNA levels between DBS eluates and corresponding plasma samples was 2.7 log_10_ IU/mL. Fifteen DBS eluates positive for HBV DNA were sequenced and all of them belonged to HBV genotype A. Thirteen samples which were negative for all HBV markers showed HBeAg false positivity. Therefore, DBS is a reliable sample matrix for detection of HBsAg, total anti-HBc and HBV DNA, but not HBeAg. Further feasibility studies of DBS for diagnostic purposes and epidemiologic studies are warranted.

**Supplementary Information:**

The online version contains supplementary material available at 10.1186/s13104-022-06178-x.

## Introduction

The World Health Organization reported that, in 2015, approximately 257 million people were still living with hepatitis B virus (HBV) infection [1]. HBV causes both acute and chronic infections with potential to induce malignant transformation to hepatocellular carcinoma. Hepatitis B ranked 15th among all causes of human mortality [2, 3]. HBV infection is prevalent in African (6.1%) and Western Pacific (6.2%) populations [1]. The diagnosis for HBV infection is based on detection of various markers which include hepatitis B surface antigen (HBsAg), total hepatitis B core antibody (total anti-HBc), hepatitis B e antigen (HBeAg), and HBV DNA. The sample matrices for these markers are generally serum and plasma samples, which require phlebotomy and subsequent pre-analytic processing of blood samples. Phlebotomy, in addition to being more invasive, labor intensive and incurs higher costs in cold chain during transportation of serum or plasma samples. Dried blood spots (DBS), on the other hand, provide an attractive alternative that obviate these requirements of phlebotomy, cold chain conditions and additional associated costs.

Obtaining DBS is much less invasive than drawing blood, since it only needs finger-pricking for sampling. Additionally, it doesn’t require subsequent centrifugation, refrigeration and large space for shipment and storage, which contribute to high costs. Thus, these advantages of DBS expedite large-scale serologic studies. DBS has been successfully used for detection of various markers of neonatal, congenital, metabolic disorders and infectious diseases [4, 5].

The potential use of DBS, as an alternative sample material, for the detection of HBV markers of infection has been explored [6–10]. The aim of this study was to further evaluate the feasibility of using DBS for detection of HBsAg, total anti-HBc, HBeAg, and HBV DNA.

## Main text

### Materials and methods

#### Engineered DBS

Positive and negative control DBS samples were engineered by spotting reconstituted blood consisting of 60% of plasma and 40% of red blood cells (RBC) on filter paper, using the protocol as previously described [4] (Additional file 1: Fig. S1). We prepared engineered DBS and tested them after storing for 1–30 days at − 20 °C. Fifty-four well characterized plasma samples previously tested for all hepatitis markers [11] and O + RBC (Tennessee Blood Services, Memphis, TN, US) were used to create the engineered DBS. The serological profiles of the samples are shown in Table 1. For each of the markers, the panel included 9 samples positive for total anti-HBc, 25 samples positive for HBsAg, 25 samples positive for HBV DNA. Twenty-eight samples negative for all HBV markers were included as negative controls. Among 54 plasma samples, 20 samples including 5 samples with high HBV DNA titer (over 6.4 log_10_ IU/mL), 3 samples with middle (4.0–6.4 log_10_ IU/mL), 2 samples with low (2.0–4.0 log_10_ IU/mL) and 10 samples negative for HBV DNA were selected to test for HBeAg. Of these samples, 7 samples were detected to be positive and 13 samples were negative. For quantitative determination of HBV DNA levels in plasma and corresponding DBS eluates, a standard curve was generated by tenfold serial dilutions of AccuSpan™ HBV DNA Linearity Panel (SeraCare, Milford, MA, US) combined with the RBC (titers ranged from 2.7 log_10_ IU/mL to 7.7 log_10_ IU/mL) to make the standard curve spots.

**Table 1:** Serological profile of 54 test and control samples used for evaluation of DBS for detection of HBV markers of infection

Total anti-HBc	HBsAg	HBV DNA	HBeAg	N = 54
Pos	Pos	Pos	Pos	2
Pos	Pos	Pos	Neg	6
Pos	Neg	Neg	Neg	1
Neg	Pos	Pos	Pos	5
Neg	Pos	Pos	Neg	12
Neg	Neg	Neg	Neg	28

Pos: positive, Neg: negative

#### DBS elution

Three spots (containing approximately 50 μL of blood per spot) were collected from each sample by punching disks using a hole-puncher to completely excise the entire blood spot. These disks were transferred directly into an Eppendorf tube and 1.2 mL of phosphate buffer saline (PBS) was added to it for elution resulting in an estimated plasma dilution of 3:40. Elution of the DBS was carried out at 56 °C in a shaker at 700 rpm for 1 h. Eluates were collected in new Eppendorf tubes after the disks had turned white, indicating complete elution of samples. DBS eluates were immediately tested for HBsAg, total anti-HBc, HBeAg and HBV DNA.

#### Detection of HBV markers

The engineered DBS with corresponding plasma samples were tested for HBsAg and total anti-HBc using chemiluminescent microparticle immunoassay (CMIA) in ARCHITECT i2000SR (04P5325 and 06L2225, Abbott Diagnostics, Abbott Park, Chicago, IL, US), following the manufacturer’s instructions.

HBeAg was tested by enzyme-linked immuno sorbent assay (ELISA) using ETI-EBK PLUS (DiaSorin, Saluggia, VC, Italy) and chemiluminescence assay in VITROS ECi Immunodiagnostic System (Ortho-Clinical Diagnostics, Inc., Rochester, NY, US).

For detection of HBV DNA from plasma and DBS eluate samples, total nucleic acids were extracted from 200 μL of samples using the MagNApure Total Nucleic Acid Isolation Kit (Roche Applied Science, Rochester, NY, US) on the MagNApure LC instrument (Roche Applied Science, Indianapolis, IN, US) with a final elution volume of 50 μL. The dual-labeled Taqman HBV probe and HBV primers were designed from an alignment of representative S-gene sequences of all HBV genotypes as previously described [12]. Detection of HBV DNA by quantitative polymerase chain reaction (qPCR) was performed using 10 μL of extracted nucleic acids in a total reaction volume of 25 μL with Express Supermix (Life Technologies, Grand Island, NY, US). The LC480 system (Roche Applied Science, Rochester, NY, US) was used for amplification, detection, and data analysis. Cycle numbers of amplification were increased to 55 cycles to optimize the condition for getting more PCR products, considering the use of DBS. After uracil-DNA glycosylase activation and incorporation, there was a denaturation step at 95 °C for 2 min. Amplification was conducted by 55 cycles of 95 °C for 10 s, 60 °C for 60 s, and with cooling at 40°C for 30 s. A standard curve was created by using the DBS engineered with tenfold serial dilutions of AccuSpan™ HBV DNA Linearity Panel (SeraCare, Milford, MA, US) as described above.

All HBV DNA positive samples were re-amplified for sequencing by a nested Sybr qPCR targeting the S gene, using primers S1F and S1R in the primary reaction and SNF and SNR in the nested reaction (Additional file 2: Table S1). The first-round PCR conditions included incubation at 94 °C for 6 min, followed by 35 cycles of 94 °C for 45 s, 55 °C for 20 s, 72 °C for 60 s, 72 °C for 7 min. Nested PCR was started at 94 °C for 6 min, followed by 30 cycles of 94 °C for 45 s, 55 °C for 30 s, 72 °C for 60 s, and 72 °C for 7 min. Sequencing reactions were conducted using the BigDye v2.1 chemistry sequencing kit (Life Technologies, Grand Island, NY, US), and products were sequenced using the 3130xl Genetic Analyzer (Life Technologies, Grand Island, NY, US). Sequencing was done in both the forward and reverse directions with internal nested primers. Sequencing PCR involved 25 cycles, each cycle consisting of 96 °C for 10 s, 50 °C for 5 s and 60 °C for 2 min [12]. All PCR programs above include a hold step at 4 °C at the end. Sequence analysis was conducted using SeqMan Pro (DNASTAR, Inc., Madison, WI, US) and phylogenetic tree was created by MEGA 7.0.26 [13].

#### Reproducibility of serological tests and qPCR

For serological tests, reproducibility was confirmed by testing two HBsAg positive (Signal-to-cut off ratio (S/CO) of 2232.1 and 1762.9), two total anti-HBc positive (S/CO of 10.5 and 10.4) and two both negative samples. HBeAg testing was repeated with all 20 samples which were selected for the test as mentioned above. For molecular test, HBV DNA high, medium, low titer and negative samples were selected: 8.2 log10 IU/mL, 6.2 log10 IU/mL, and 4.4 log10 IU/mL. All Samples were tested in triplicate by the same operator for three days using the same process.

### Results

For engineered DBS, sensitivity, specificity and percent agreement between DBS and corresponding plasma were determined by comparing the results of HBsAg, total anti-HBc, HBeAg and HBV DNA in DBS eluates to the results of their corresponding plasma samples (sensitivity (%) = number of positive samples in engineered DBS/number of positive samples in plasma, specificity (%) = number of negative samples in engineered DBS/number of negative samples in plasma, percent agreement (%) = number of samples which concordantly represent positive or negative results in plasma and engineered DBS/number of plasma samples). The sensitivity, specificity and percent agreement of HBsAg detection in DBS was 96.0%, 100% and 98.1%, respectively. One sample that showed discordant result between DBS and corresponding plasma had a lower (6.4) S/CO in corresponding plasma sample (Table 1). Total anti-HBc was detected in all 9 DBS eluates indicating a 100% sensitivity, specificity and percent agreement with corresponding plasma samples (Table 1). HBeAg was detected in all 7 DBS eluates whose corresponding plasma samples were also HBeAg positive, however, HBeAg was also detected in all of 13 samples whose corresponding plasma samples were HBeAg negative (Table 1).

**Fig. 1:**
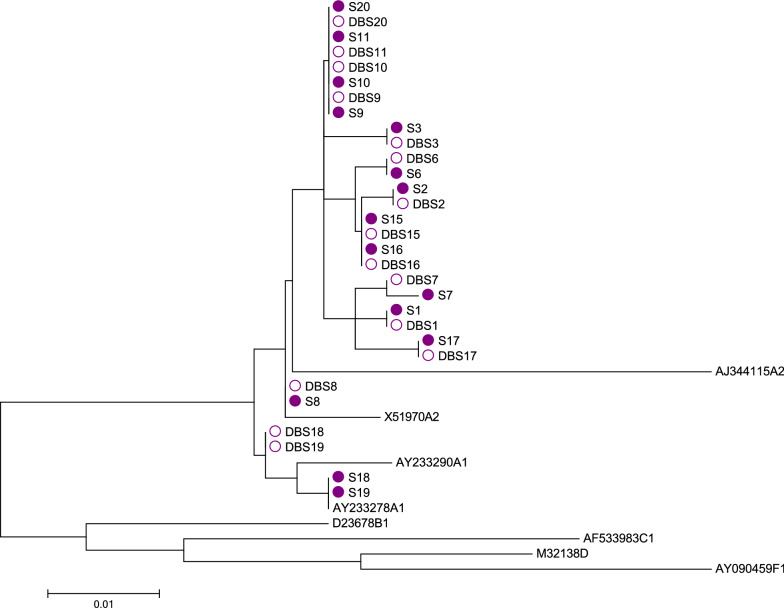
Phylogenetic tree of engineered dried blood spots (DBS, hollow bullets) and corresponding plasma (colored bullets); sequences without a bullet represent reference. Hepatitis B virus (HBV) DNA sequences of 15 samples are all concordant between DBS and corresponding plasma and are determined to be genotype A

HBV DNA detection showed 88.0% of sensitivity, 100% of specificity and 94.4% of percent agreement (Table 1). The three DBS samples from which HBV DNA was undetectable had 2.47, 2.61 and 2.85 log_10_ IU/mL HBV DNA (average: 2.64 log_10_ IU/mL) in their corresponding plasma samples (Table 2). The limit of detection (LOD) of HBV DNA titer in engineered DBS was 2.0 log_10_ IU/mL and the average of HBV DNA titer difference between DBS and corresponding plasma was 2.7 log_10_ IU/mL. From engineered DBS and corresponding plasma, HBV DNA was sequenced in 15 of 22 (68.0%) samples. Sequences were concordant between those of DBS and corresponding plasma samples, and all were determined to be genotype A (Fig. 1).

**Table 2:** Result of HBsAg, total anti-HBc, HBeAg, HBV DNA detection in engineered Dried Blood Spots (DBS) and corresponding plasma samples

	Plasma			Engineered DBS		Sensitivity (%)	Specificity (%)	Percent Agreement (%)
	Positive	Negative		Positive	Negative			
HBsAg	25	29		24	29	96.0	100	98.1
anti-HBc	9	45		9	45	100	100	100
HBeAg	7	13		20	0	100	0	35.0
HBV DNA	25	29		22	29	88.0	100	94.4

anti-HBc: hepatitis B core antibody, DBS: dried blood spot, HBeAg: hepatitis B e antigen, HBsAg: hepatitis B surface antigens, HBV: hepatitis B virus

Reproducibility was confirmed to be 100% in HBsAg, total anti-HBc, HBeAg and HBV DNA testing with DBS.

### Discussion

In this study we evaluated the use of DBS samples for detection of HBsAg, HBV DNA, total anti-HBc and HBeAg using well characterized plasma samples and their corresponding engineered DBS samples. Three markers except HBeAg were detected in DBS eluates with 100% concordance for total anti-HBc, 98% for HBsAg and 94% for HBV DNA despite a much smaller sample volume and its dilution during the elution process from DBS. The sensitivity of total anti-HBc in DBS in our study far exceeds the lower sensitivities reported in several previous studies (range 68.0% to 90.5%, average 81.2%) [14–18], although the sample input volume and dilution factors were similar in our study and these studies [16]. It is possible that the protocol for elution of samples from DBS used in our study results in a better yield of the sample and using freshly eluted samples for testing further contributes to a better sensitivity. Detection of HBsAg from DBS also demonstrated excellent performance, which were similar to the report of latest systematic review (98% of sensitivity and 100% of specificity) [7, 9].

The sensitivity of HBV DNA was lower than that of HBsAg and total anti-HBc (88%). This might be explained by loss of HBV DNA titer due to small volume of DBS samples and potential degradation of DNA through multiple steps of DBS processing, such as elution or extraction procedures. The LOD of HBV DNA was 2.0 log_10_ IU/mL and the average of HBV DNA titer difference between DBS and corresponding plasma was 2.7 log_10_ IU/mL in this study. According to the latest systematic review on HBV DNA quantification using DBS, the lowest limit of detection was around 3.0 log_10_ IU/mL [8, 9], which suggests inevitable HBV DNA titer loss in DBS with the current methods. Despite the limitation with the detection of HBV DNA in DBS, as a screening tool for large population studies, the majority of patients currently infected by HBV will be able to be identified. However, unreliable results were observed in HBeAg detection in our study. To date, two studies examined the utility of HBeAg testing with DBS [19, 20]. Mohamed et al. got consistent results of 10 HBeAg positives in both plasma and DBS by immunoassay (No testing platform detail was provided) among 100 HBsAg positive samples [20]. Jackson et al. could detect HBeAg positivity with 80.9% of sensitivity (17/21 samples) by chemiluminescence technology using LIAISON XL (Diasorin, Saluggia, Italy) among 98 HBsAg positive samples [19]. False positivity was not observed in these studies. According to our results, however, HBeAg negative samples concordantly showed false positive in DBS when these were tested by ELISA using ETI-EBK PLUS (DiaSorin) and chemiluminescence technology using VITROS ECi Immunodiagnostic System (Ortho-Clinical Diagnostics, Inc.). The factors responsible for this false positivity for HBeAg remain to be determined.

Use of DBS samples also allows sequencing of viral genome and subsequent determination of viral genotype. Ability to generate sequences from HBV DNA positive DBS eluates is an additional advantage of using DBS.

## Conclusion

DBS can be a reliable sample matrix for detecting markers of HBV infection in large population studies, especially in resource-limited countries. Three key markers of HBV infection, HBsAg, total anti-HBc and HBV DNA, were detected in DBS with high accuracy in our study. Further studies are needed for elucidating and evaluating the feasibility of DBS for HBeAg testing and optimization of elution protocols to improve the sensitivity of HBV DNA detection.

## Limitations

Our study has some limitations. First, we used only engineered DBS samples. Paired DBS and plasma samples from clinical studies were not available for further validation of DBS. Second, only two platforms, ETI-EBK PLUS and VITROS ECi Immunodiagnostic System, were used for HBeAg testing. Further trials with other platforms are essential to elucidate why DBS samples showed false positivity.

## Supplementary Information

**Additional file 1: Figure S1.** Protocol of engineering dried blood spots (DBS) [4]. Use 850 mL of whole blood with O+ red blood cells (RBC) and wash the RBC with 50 mL of 0.9% saline to remove plasma, buffy coat, and anticoagulant. Centrifuge the mixture of whole blood and 0.9% saline at 4,000 rpm in 21 °C for 8 min and discard the supernatant. Repeat the wash procedure three times, and centrifuge for 15 min at the last time. Then confirm the hematocrit of RBC is at least 95%. To reconstitute the whole blood (40% of RBC and 60% of plasma) using the washed RBC, 1 mL of RBC and 1.36 mL of well characterized plasma samples are mixed thoroughly. The reconstituted whole blood is spotted onto filter paper at 50 µL blood per spot.

**Additional file 2: Table S1. **Primers and probes of hepatitis B virus (HBV) DNA test and sequencing.

## Data Availability

The datasets used and/or analysed during the current study are available from the corresponding author on reasonable request.

## Abbreviations

DBS: Dried blood spot
HBeAg: Hepatitis B e antigen
HBsAg: Hepatitis B surface antigens
HBV: Hepatitis B virus
Total anti-HBc: Total hepatitis B core antibody
