# Supplementary material for: Dried blood spot is the feasible matrix for detection of some but not all hepatitis B virus markers of infection
Source: BMC Res Notes. 2022 Sep 5;15:287. doi: 10.1186/s13104-022-06178-x (PMC9446784; doi:10.1186/s13104-022-06178-x)
Supplement: Supplementary file 1 — Additional file 1: Figure S1. Protocol of engineering dried blood spots (DBS) [4]. Use 850 mL of whole blood with O+ red blood cells (RBC) and wash the RBC with 50 mL of 0.9% saline to remove plasma, buffy coat, and anticoagulant. Centrifuge the mixture of whole blood and 0.9% saline at 4,000 rpm in 21 °C for 8 min and discard the supernatant. Repeat the wash procedure three times, and centrifuge for 15 min at the last time. Then confirm the hematocrit of RBC is at least 95%. To reconstitute the whole blood (40% of RBC and 60% of plasma) using the washed RBC, 1 mL of RBC and 1.36 mL of well characterized plasma samples are mixed thoroughly. The reconstituted whole blood is spotted onto filter paper at 50 µL blood per spot. [file 13104_2022_6178_MOESM1_ESM.pptx]

## Slide 1
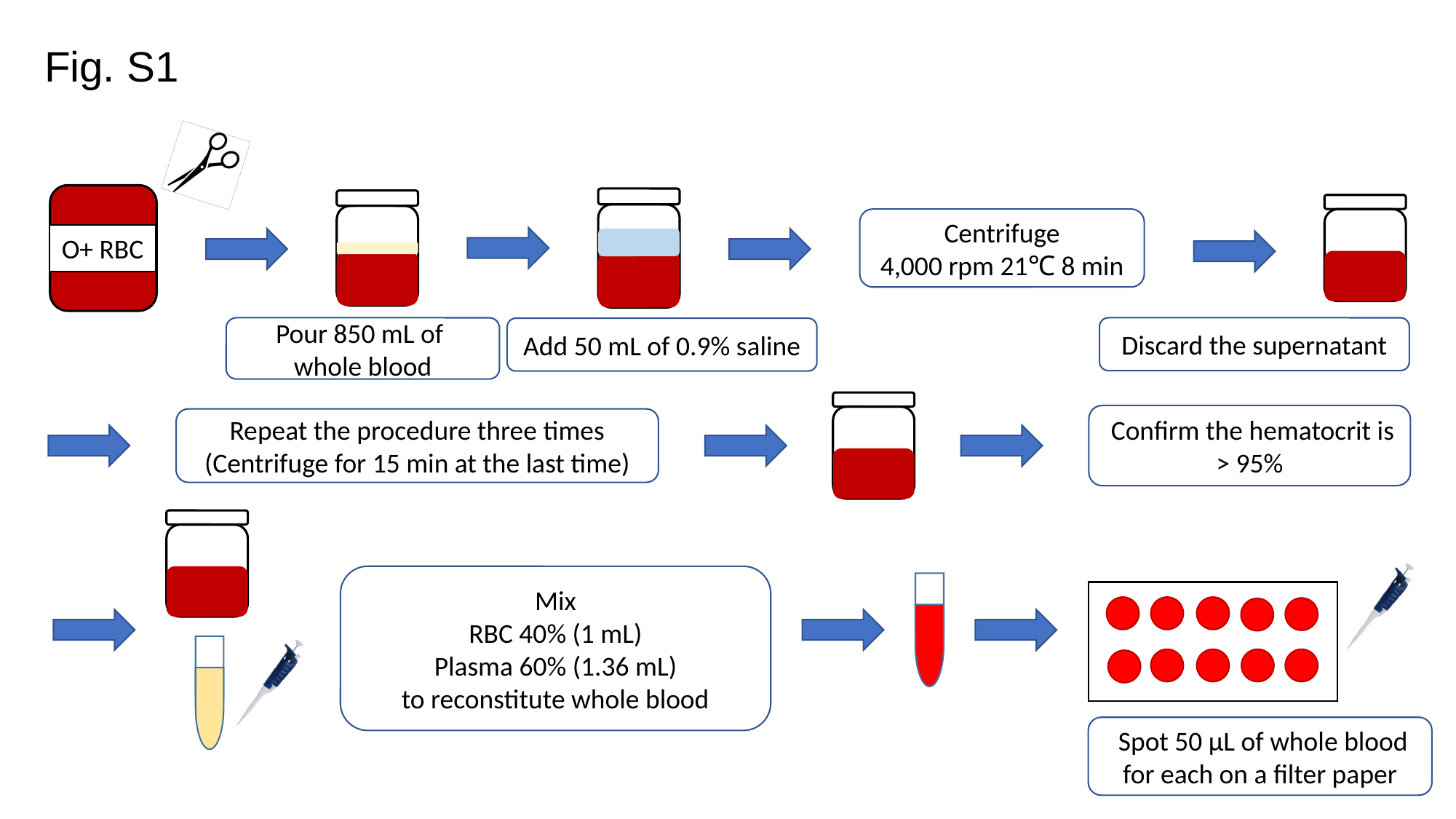

Fig. S1
O+ RBC
Centrifuge
4,000 rpm 21℃ 8 min
Pour 850 mL of
whole blood
Discard the supernatant
Add 50 mL of 0.9% saline
 Confirm the hematocrit is > 95%
Repeat the procedure three times
(Centrifuge for 15 min at the last time)
Mix
RBC 40% (1 mL)
Plasma 60% (1.36 mL)
to reconstitute whole blood
 Spot 50 µL of whole blood for each on a filter paper
