# Supplementary material for: Dried blood spot is the feasible matrix for detection of some but not all hepatitis B virus markers of infection
Source: BMC Res Notes. 2022 Sep 5;15:287. doi: 10.1186/s13104-022-06178-x (PMC9446784; doi:10.1186/s13104-022-06178-x)
Supplement: Supplementary file 2 — Additional file 2: Table S1. Primers and probes of hepatitis B virus (HBV) DNA test and sequencing. [file 13104_2022_6178_MOESM2_ESM.docx]

**Table S1.**

**Primers and probes of hepatitis B virus (HBV) DNA test and sequencing**

| Primer ID | Sequence |
| --- | --- |
| HBV 384P | 6FAM TGC GGC GTT TAT CAT MTT CCT CTT CAT-BHQ |
| HBV 395F | TGT CCT GGY TAT CGC TGG AT |
| HBV 425R | CCA ACA AGA AGA TGA GGC ATA GC |
| S1F | CTAGGACCCCTGCTGGTGTT |
| S1R | TCGAACCACTGAACAAATGGCACT |
| SNF | GTTGACAAGAATCCTCACAATACC |
| SNR | GGCTGAGGCCCACTCCCATA |

The same primers and probes were used as those reported by Tonya Mixon-Hayden [12].
